# Effects of berberine on the pharmacokinetics of florfenicol and levels of cytochrome P450 3A37, multidrug resistance 1, and chicken xenobiotic‐sensing orphan nuclear receptor mRNA expression in broilers

**DOI:** 10.1002/vms3.660

**Published:** 2021-11-11

**Authors:** Sicong Li, Bin Wang, Min Zhang, Dingsheng Yuan, Jinliang Li, Xuting Li, Ge Liang

**Affiliations:** ^1^ Sichuan Animal Science Academy Chengdu P. R. China; ^2^ Animal Breeding and Genetics Key Laboratory of Sichuan Province Chengdu P. R. China; ^3^ Sichuan Dingjian Animal Pharmaceutical Co., Ltd. Chengdu P. R. China

**Keywords:** berberine, cytochrome P450, florfenicol, multidrug resistance 1, pharmacokinetics

## Abstract

**Background:**

Berberine (BBR) is always used in combination with florfenicol for treating avian in China.

**Objective:**

This study aims to investigate the effects of BBR on the pharmacokinetics of florfenicol in broilers.

**Methods:**

Male broilers were randomly divided into the control group and the BBR group (BG). Note that 50 mg/kg BBR or sterile water was orally administrated to broilers. On the 8th day, florfenicol [30 mg/kg body weight (BW)] was orally administered to broilers in both groups. The plasma concentrations of florfenicol were determined by ultra‐high‐performance liquid chromatography (UHPLC). The levels of cytochrome P450 (CYP) 3A37, multidrug resistance 1 (MDR1), and chicken xenobiotic‐sensing orphan nuclear receptor (CXR) mRNA expression in the liver and jejunum were determined by the real‐time PCR.

**Results:**

The results showed that the C_max_, t_1/2z_, MRT_(0‐∞)_, and AUC_(0‐∞)_ of florfenicol in BG were significantly increased (by 55.71%, 28.32%, 35.19%, and 55.62%, respectively), while the T_max_ and CLz/F of florfenicol were significantly decreased (by 52.13% and 35.82%, respectively). In BG, the levels of CYP3A37, MDR1, and CXR mRNA expression in the liver were significantly decreased to 0.72‐fold, 0.67‐fold, and 0.59‐fold, respectively, and the corresponding mRNA expression in the jejunum were significantly decreased to 0.66‐fold, 0.55‐fold, and 0.64‐fold levels, respectively, relative to their levels in the control group.

**Conclusions:**

BBR altered the pharmacokinetics of florfenicol, probably related to its inhibition of CYP3A37, MDR1, and CXR mRNA expression in the jejunum and liver.

## Graphical abstract

We investigated the effects of berberine on the pharmacokinetics of florfenicol in broilers. The plasma concentrations of florfenicol in broilers with or without BBR pre‐treatment were determined by UHPLC method. In addition, the effects of berberine on CYP3A37, MDR1, and CXR mRNA expression in broilers were determined by real‐time PCR. The results indicated that berberine altered the pharmacokinetics of florfenicol, probably be related to its inhibition of CYP3A37, MDR1, and CXR mRNA expression in the jejunum and liver.

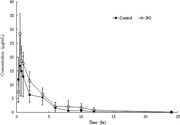

## 1 INTRODUCTION

**FIGURE 1:**
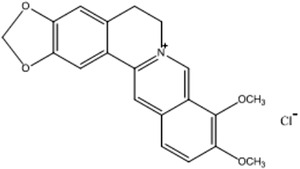
Chemical structure of berberine

Berberine (BBR) (Figure 1) is a bioactive herbal ingredient isolated from many herb families, such as *Berberis aristata* and *Coptis chinensis*. It has a variety of pharmaceutical effects, including effects on gastroenteritis and secretory diarrhoea, and is safe for use in humans (Luo et al., 2013). BBR also has a good inhibitory effect on drug‐resistant strains. X. Li et al. (2021) reported that BBR hydrochloride could reverse the resistance of multidrug‐resistant *Acinetobacter baumannii* to tigecycline, sulbactam, meropenem, and ciprofloxacin. Shi et al. (2018) indicated that the combination of BBR and ciprofloxacin has a synergistic antibiofilm effect on multi‐resistant *Salmonella* via inhibiting the mRNA expressions of luxS, rpoE, and ompR. In Chinese veterinary clinics, it is commonly used to prevent and treat diarrhoea in chickens (Shen et al., 2010). Previous studies showed that BBR can decrease human cytochrome P450 (CYP) 3A (Guo et al., 2012) and P‐gp activity (Zhang et al., 2019), and modulate the metabolism of midazolam and rhodamine 123 in rats (Xin et al., 2016). Therefore, it is necessary to be concerned about drug–drug interactions that might occur between BBR and drugs metabolized by cytochrome P450 enzymes and/or efflux transporters.

Florfenicol is a broad‐spectrum synthetic antibiotic commonly used in veterinary practice to control various bacterial infections (Pérez et al., 2015; Sidhu et al., 2014; Wang et al., 2013). It has the same activity profile as chloramphenicol. However, it has less toxicity and better antibacterial activity than chloramphenicol. Several reports have described the possible metabolic pathways and mechanisms of florfenicol in vivo. N. Liu et al. (2012) reported that P‐gp and/or CYP3A were likely involved in the disposition of florfenicol in rabbits. Wang et al. (2018) suggested that CYP3A may play a critical role in the metabolism of florfenicol, and P‐gp may be involved in the absorption of florfenicol in chickens. Therefore, if broilers are given both traditional Chinese medicine (TCM) preparations containing BBR and florfenicol in veterinary clinics during a specific treatment period, BBR might affect the pharmacokinetics of florfenicol by modulating P‐gp and/or CYP3A, thereby leading to drug–drug interactions between BBR and florfenicol.

Here, we investigated the effects of BBR on the pharmacokinetics of florfenicol in broilers. The plasma concentrations of florfenicol in broilers with or without BBR pre‐treatment were determined by an ultra‐high‐performance liquid chromatography (UHPLC) method, and values for pharmacokinetic parameters were calculated. In addition, the effects of BBR on CYP3A37, multidrug resistance 1 (MDR1), and chicken xenobiotic‐sensing orphan nuclear receptor (CXR) mRNA expression in broilers were determined by real‐time PCR. Our results can be used to help predict the clinical effects or risks for BBR–florfenicol interactions.

## 2 MATERIALS AND METHODS

### 2.1 Chemicals and reagents

BBR, florfenicol, and chloramphenicol analytical standards were obtained from the National Institutes for Food and Drug Control (Beijing, PR China). BBR for administration was dissolved in sterile water to a concentration of 50 mg/ml. Florfenicol was supplied by Hubei Longxiang Pharmaceutical Tech. Co., Ltd. (Huanggang, PR China) and subsequently dissolved in polyethylene glycol 400 (Kelilong, Chengdu, PR China) to a concentration of 30 mg/ml. Acetonitrile and methanol ethyl acetate (HPLC‐grade) were purchased from Merck Chemicals Co., Ltd., Darmstadt, Germany. All other chemicals used for analysis were of analytical grade.

### 2.2 Animals and treatment

A total of 24 healthy daheng broilers [male; 2.45 ± 0.15 kg body weight (BW)] were provided by Sichuan Daheng Poultry Breeding Co., Ltd. (Chengdu, PR China). Only male broilers were used in the study to minimize the impact of hormones and other physiological factors on pharmacokinetics. The broilers were allowed free access to water and a standard diet that lacked any medicine supplements. All procedures were performed in accordance with principles in the National Institutes of Health Guide for the Care and Use of Laboratory Animals. The study protocol was approved by the Ethics Committee of Sichuan Animal Science Academy (Approval No.: 2020‐011). Broilers were randomly divided into two groups: the control group and the BBR group (BG) (*n* = 12 per group). Note that 50 mg/kg BBR was orally administrated directly into the crop of each animal in the BG group by tube gavage during the morning of each day for 7 consecutive days, and the equal amounts of sterile water were administrated by gavage to broilers in the control group. On the 8th day, each group was divided into two, of which six broilers were used in pharmacodynamic study, and the other six were used to evaluate the levels of CYP3A37, MDR1, and CXR mRNA expression. During the whole trial period, there was no significant difference in feed intake, growth, and health status between the BG group and the control group.

### 2.3 Pharmacokinetic study

On the 8th day, after 12 h of fasting, florfenicol (30 mg/kg BW) was orally administered into the crop of broilers in both groups. A blood sample (approximate 0.5 ml) was aseptically collected from the wing vein of each animal into heparinized tubes at 0.25, 0.50, 0.75, 1, 2, 4, 6, 8, 10, 12, and 24 h after florfenicol administration. Plasma samples were obtained after centrifugation at 4000 rpm for 5 min and stored (−80°C) until analysis. Each plasma sample was prepared as previously described (S. Li et al., 2019). UHPLC analyses were carried out using an UltiMate 3000 HPLC system (Thermo Fisher Scientific Inc., Chelmsford, MA, USA), and the corresponding configuration of this system has been described in our previous report (S. Li et al., 2017). A Diamonsil C18 column (4.6 mm × 250 mm, 5 μm; Thermo Fisher Scientific Inc.) was used to simultaneously detect florfenicol and chloramphenicol at a constant temperature of 40°C. The mobile phase was composed of acetonitrile and water (27:73, v:v), the flow rate was 1.0 ml/min, and the ultraviolet (UV) wavelength was 223 nm. The sample injection volume was 20 μl, and the analysis time was 16 min. For florfenicol, the limit of detection (LOD) and limit of quantification (LOQ) were detected based on signal‐to‐noise ratios of 3 and 10, and they were validated at 0.05 and 0.02 μg/ml, respectively. The intra‐day and inter‐day assay precisions for 3 days at three standard levels (0.1, 2.5, and 20 μg/ml) were all less than 6.8%. The extraction recoveries of three concentrations of florfenicol all exceeded 85.2%. The *R* ^2^ value of the calibration curve was 0.9997.

### 2.4 RNA extraction and real‐time PCR

On the 8th day, after 12 h of fasting, the animals were sacrificed in a carbon dioxide asphyxiation machine. After opening the coelom, a sample of tissue was quickly ectomized from each liver and jejunum, perfused with chilled physiological saline solution to remove blood residue, and blotted dry. Small piece of each sample (approximate 1 g) was collected in a nuclease free cryogenic vial containing 1 ml of RNA‐EZ reagents (Sangon Biotech (Shanghai) Co., Ltd., Shanghai, P.R. China) and frozen at −80°C. The total RNA was extracted from each sample by using TRIzol reagent (Invitrogen Corporation and Applied Biosystems Inc., Carlsbad, CA, USA) according to the instructions provided in the user manual. RNA concentration, purity, and integrity were measured in accordance with our previous study (S. Li et al., 2017). RNA samples (0.8 μg each) were reverse transcribed to cDNA using RevertAid Premium Reverse Transcriptase (Thermo Fisher Scientific Inc.) according to the user manual. The cDNA products were frozen and stored at −80°C.

Real‐time PCR was carried out on an ABI StepOne RT‐PCR instrument (Applied Biosystems, Foster City, CA, USA) in a 20 μl final volume that contained 10 μl High RoxSybrGreen qPCR Master Mix (Sangon Biotech (Shanghai) Co., Ltd.), 2 μl cDNA, 0.4 μl of each oligonucleotide primer (10 μM), and 7.2 μl diethyl pyrocarbonate‐treated autoclaved distilled water. Real‐time PCR amplification was performed under the following conditions: initial denaturation at 95°C for 3 min, followed by 45 cycles of denaturation at 95°C for 10 s, annealing at 57°C for 15 s, extension at 72°C for 20 s, and final extension at 60°C for 1 min. The β‐actin house‐keeping gene was used as the control. Real‐time PCR data were calculated by the 2^−(∆∆^ *^Ct^* ^)^ method (Livak & Schmittgen, 2001). The sequences of the forward and reverse primers are presented in Table 1.

**TABLE 1:** Sequences of the forward and reverse primers used for real‐time polymerase chain reaction (RT‐PCR)

Isozymes	Accession number	PCR efficiency (%)	Forward	Reverse
CXR	NM_204702.1	100.792	5′‐TCCCTTCGGCATCCCTGTC‐3′	5′‐GGCGTTGGTCTCCTCGTTG‐3′
MDRl	XM_003641637.1	108.089	5′‐GCTGTTGTATTTCCTGCTATGG‐3′	5′‐ACAAACAAGTGGGCTGCTG‐3′
CYP3A37	NM_001001751.2	104.632	5′‐CGAATCCCAGAAATCAGA‐3′	5′‐AGCCAGGTAACCAAGTGT‐3′
β‐Actin	NM_205518.1	104.719	5′‐ATGTGGATCAGCAAGCAGGAGTA‐3′	5′‐TTTATGCGCATTTATGGGTTTTGT‐3′

Abbreviations: CXR, chicken xenobiotic‐sensing orphan nuclear receptor; MDRl, multidrug resistance 1.

### 2.5 Statistical analysis

Data Analysis System software (Version 3.0; Chinese Pharmacological Society, Beijing, PR China) was used to calculate values for the pharmacokinetic parameters of florfenicol. A noncompartmental method was used to determine the area under the concentration–time curve (AUC), mean residence time (MRT), the elimination half‐life (*t*_1/2z_), the plasma clearance fraction of the dose absorbed (CLz/F) and the apparent volume of distribution fraction of the dose absorbed (Vz/F). The peak concentration (*C*_max_) and the time to reach peak concentration (*T*_max_) were directly obtained.

All data are presented as mean ± SD. Statistically significant values were calculated by one‐way analysis of variance (ANOVA), performed using IBM SPSS Statistics for Windows, version 22.0 (IBM Corp, Armonk, NY, USA). In all tests, a *p*‐value < 0.05 was considered to be statistically significant.

## 3 RESULTS

### 3.1 Effect of BBR on the pharmacokinetics of florfenicol

**FIGURE 2:**
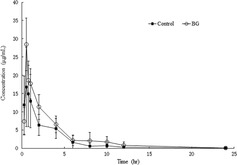
Mean plasma concentration–time profiles of florfenicol in broilers after oral administration of florfenicol [30 mg/kg body weight (BW)] with or without pre‐treatment with berberine (BBR) (50 mg/kg BW for 7 days). Each symbol with a bar represents the mean value ± SD for six broilers

The effects of BBR on the pharmacokinetics of florfenicol in broilers are shown in Table 2 and Figure 2. After the broilers were pre‐treated with BBR for 7 consecutive days, the values for AUC_(0‐∞)_, as well as the values for MRT_(0‐∞)_, *t*_1/2z_, and *C*_max_ of florfenicol in the BG group were significantly increased by 55.62%, 35.19%, 28.32%, and 55.71%, respectively, when compared with their values in the control group. However, the values for *T*_max_ and CLz/F were significantly decreased by 52.13% and 35.82%, respectively, when compared with their values in the control group. In addition, the values for Vz/F in the BG group were not significantly different from those in the control group.

**TABLE 2:** Pharmacokinetic characteristics of florfenicol in the plasma of broilers with or without berberine (BBR) pre‐treatment

Characteristic	Control	BG
AUC_(0‐∞)_ (mg/L × h)	46.85 ± 11.89	72.91 ± 15.33*
MRT_(0‐∞)_ (h)	2.87 ± 0.35	3.88 ± 0.57*
*t*_1/2z_ (h)	2.79 ± 0.34	3.58 ± 0.58*
*T*_max_ (h)	0.94 ± 0.43	0.45 ± 0.11*
Vz/F (L/kg)	2.38 ± 1.24	2.42 ± 1.96
CLz/F (L/h/kg)	0.67 ± 0.14	0.43 ± 0.11*
*C*_max_ (mg/L)	18.58 ± 4.66	28.93 ± 5.57*

*Note*: Pharmacokinetic characteristics of florfenicol in the plasma of broilers after oral administration of florfenicol (30 mg/kg BW) with or without BBR (50 mg/kg BW for 7 days) pre‐treatment (*n* = 6, mean ± SD).

Abbreviation: BG, BBR group.

* Significantly different from the control group*, p* < 0.05.

### 3.2 Effect of BBR on CYP3A37, MDR1, and CXR mRNA expression in the liver and jejunum

**FIGURE 3:**
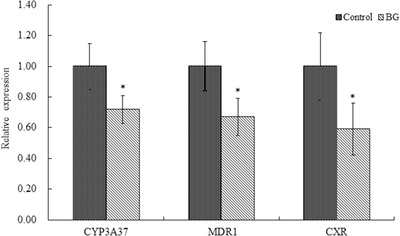
Effect of berberine [BBR; 50 mg/kg body weight (BW) for 7 days] on CYP3A37, multidrug resistance 1 (MDR1), and chicken xenobiotic‐sensing orphan nuclear receptor (CXR) mRNA expression in the liver (*n* = 6). *Significantly different from the control group, *p* < 0.05

**FIGURE 4:**
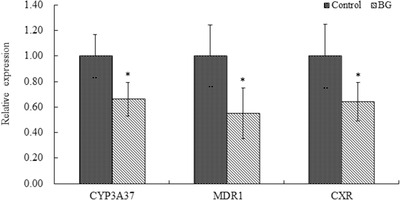
Effect of berberine [BBR; 50 mg/kg body weight (BW) for 7 days] on CYP3A37, multidrug resistance 1 (MDR1), and chicken xenobiotic‐sensing orphan nuclear receptor (CXR) mRNA expression in the jejunum (*n* = 6). *Significantly different from the control group, *p* < 0.05

The effects of BBR on CYP3A37, MDR1, and CXR mRNA expression in the liver are shown in Figure 3. After the broilers were pre‐treated with BBR for 7 consecutive days, the levels of CYP3A37, MDR1, and CXR mRNA expression in the BG group were significantly decreased to 0.72‐fold, 0.67‐fold, and 0.59‐fold, respectively, relative to their levels in the control group. The effects of BBR on CYP3A37, MDR1, and CXR mRNA expression in the jejunum are shown in Figure 4. After the broilers were pre‐treated with BBR for 7 consecutive days, the levels of CYP3A37, MDR1, and CXR mRNA in the BBR group were significantly decreased to 0.66‐fold, 0.55‐fold, and 0.64‐fold levels, respectively, relative to their levels in the control group.

## 4 DISCUSSION

The veterinary clinical dose of BBR on broilers is about 30–100 mg/kg/day, and the administration cycle is about 3–10 days. Thus, in the present study, we chose 50 mg/kg BBR for 7 consecutive days of treatment to investigate its effect on the pharmacokinetics of florfenicol in broilers and the underlying mechanism for that effect in vivo. Our results suggest that BBR alters the pharmacokinetics of florfenicol, resulting in higher plasma concentrations of florfenicol, and the effect is probably related to CYP3A37, MDR1, and CXR expression in the jejunum and liver.

In this study, 7 days of oral BBR administration increased the *C*_max_ values of florfenicol in BBR‐treated broilers and decreased the corresponding *T*_max_ values. We propose that these changes may result from BBR increasing the intestinal absorption of florfenicol. In addition, the CLz/F values of florfenicol were significantly decreased after oral administration of BBR, while the corresponding MRT_(0‐∞)_ and *t*_1/2z_ values were significantly increased, indicating that florfenicol metabolism in the liver was accelerated. We speculate that BBR decreased the elimination of florfenicol and increased its intestinal absorption, ultimately leading to increased AUC_(0‐∞)_ values for florfenicol in the BG group.

P‐gp is an important transport protein encoded by the MDR gene 1. It acts together with CYP3A to inhibit drug transport and absorption in the intestine (Lo & Burckart, 1999; Pal & Mitra, 2006). In addition, P‐gp may also be involved in florfenicol absorption in chickens (Wang et al., 2018). In the liver, P‐gp is mainly expressed on the canalicular membrane of hepatocytes, where it facilitates the transport of endogenous and exogenous substrates (Tian et al. 2007). CYP3A is a phase I metabolic enzyme and may play a key role in the metabolism of florfenicol in rabbits (N. Liu et al., 2012) and chickens (Wang et al., 2018). As members of the CYP3A gene family, the CYP3A37 and CYP3A80 genes are expressed in birds (Watanabe et al., 2013), and CYP3A37 is thought to be the major enzyme in florfenicol metabolism (Wang, 2012). Therefore, we further studied the effects of BBR on MDR1 and CYP3A37 mRNA expression. We found that the mRNA levels for jejunal MDR1 and CYP3A37 in the BBR‐treated broilers were significantly decreased, which may have reduced P‐gp and CYP3A37 activity and led to a decrease in florfenicol efflux and a corresponding increase in florfenicol absorption. This notion is consistent with the changes we observed in florfenicol pharmacokinetic properties. In addition, after oral administration of BBR, the levels of hepatic CYP3A37 and MDR1 mRNA expression were also significantly decreased, which may have reduced the levels of CYP3A37 and P‐gp activity, and thereby inhibited the metabolism and efflux of florfenicol in the liver. Such a reduction in florfenicol elimination would also be consistent with our pharmacokinetic results, which showed that the values for CLz/F were significantly decreased and the *t*_1/2z_ values for florfenicol were significantly increased. However, the activities of the corresponding drug‐metabolizing enzyme and efflux transporter were not measured in our current study, which makes it impossible to fully support the above inference. Therefore, the validity of this inference requires further investigation.

There are few reports concerning the effect of BBR on P‐gp expression and activity in chickens. Zhang et al. (2019) reported that BBR could increase the absorption of P‐gp substrates by down‐regulating P‐gp expression and activity in chicken tissues, and our study results tended to agree with that finding. Previous reports speculated that in addition to inhibiting P‐gp mRNA expression, BBR may also affect P‐gp ATPase activity, compete with other P‐gp substrates, and thus inhibit the transport function of P‐gp (Hamabe et al., 2006; Zhang et al., 2019). These characteristics and effects may help to explain why absorption of florfenicol in the jejunum of broilers was accelerated after the birds had been pre‐treated with BBR. To our knowledge, our current study is the first to show that BBR down‐regulated the levels of CYP3A mRNA expression in chickens, and this finding is in partial agreement with previous studies conducted in humans (Guo et al., 2011), rats (Xin et al., 2004), and HepG2 cell (Tang, 2015). However, a previous study conducted with HepG2 cells showed that BBR at a concentration of 1000 ng/ml could increase the levels of CYP3A4 and CYP1A2 mRNA and protein expression (Cui et al., 2014). The differences in these results may be due to differences in dosages, durations of exposure, and model species.

CXR, the main xenobiotic‐sensing nuclear receptor in chickens, is related to the pregnane X receptor (PXR) and constitutive androstane receptor (CAR) in humans, and CYP450 and MDR1 are regulated by CXR (Handschin et al., 2000, 2001). It has been reported that BBR affects P‐gp expression by regulating PXR/CAR expression (Slosky et al., 2013). In this study, the decreases in the levels of MDR1 mRNA expression in BBR‐treated animals were correlated with the decrease in CXR mRNA expression, which supports the above viewpoint.

The reported minimum inhibitory concentrations (MICs) of florfenicol for bacteria isolated from different animal species range from 0.4 to 8 μg/ml (Gharaibeh et al., 2010; Illambas et al., 2013; Lei et al., 2018; Salmon & Watts, 2000), and those bacteria display sensitivity to antibiotics at an average blood concentration 2‐4‐fold their MIC (C. Liu et al., 2015). Therefore, we speculated that the effective plasma concentration of florfenicol against various bacterial pathogens in vivo might be in the range of 1–16 μg/ml. In this study, the mean plasma concentration of florfenicol in the BG group exceeded 1 μg/ml for approximately 12 h, while that time period in the control group was only approximately 7 h (Figure 2). This observation suggests that feeding of BBR may increase the prophylactic or therapeutic effectiveness of florfenicol.

## 5 CONCLUSIONS

In this study, BBR affected the pharmacokinetics of florfenicol in broilers, probably related to its inhibition of CYP3A37, MDR1, and CXR mRNA expression in the jejunum and liver. These findings indicate that BBR may increase the bioavailability of florfenicol, and thus increase the therapeutic effect of florfenicol. The results of these studies in broilers should prompt additional studies on the effects of BBR when used in conjunction with various other pharmaceutical agents in veterinary medicine.

## CONFLICT OF INTEREST

All authors declare no conflict of interest.

## AUTHOR CONTRIBUTIONS

Sicong Li: Data curation, Formal analysis, Methodology, Writing‐original draft, Writing‐review & editing; Bin Wang: Formal analysis, Validation; Min Zhang: Data curation, Formal analysis; Dingsheng Yuan: Data curation; Jinliang Li: Validation; Xuting Li: Methodology; Ge Liang: Funding acquisition, Investigation, Project administration, Resources, Supervision.

## ETHICS STATEMENT

The ethics committee of Sichuan Animal Science Academy approved all experiments.

## Data Availability

Data available by permission to authors.

## References

[vms3660-bib-0002] Cui, H. M. , Zhang, Q. Y. , Wang, J. L. , Chen, J. L. , Zhang, Y. L. , & Tong, X. L. (2014). In vitro studies of berberine metabolism and its effect of enzyme induction on HepG2 cells. Journal of Ethnopharmacology, 158, 388–396. 25456436 10.1016/j.jep.2014.10.018

[vms3660-bib-0009] Gharaibeh, S. , Al Rifai, R. , & Al‐Majali, A. (2010). Molecular typing and antimicrobial susceptibility of *Clostridium perfringens* from broiler chickens. Anaerobe, 16(6), 586–589. 20969969 10.1016/j.anaerobe.2010.10.004

[vms3660-bib-0003] Guo, Y. , Pope, C. , Cheng, X. , Zhou, H. , & Klaassen, C. D. (2011). Dose–response of berberine on hepatic cytochromes P450 mRNA expression and activities in mice. Journal of Ethnopharmacology, 138(1), 111–118. 21920422 10.1016/j.jep.2011.08.058 PMC3384737

[vms3660-bib-0004] Guo, Y. , Chen, Y. , Tan, Z. R. , Klaassen, C. D. , & Zhou, H. H. (2012). Repeated administration of berberine inhibits cytochromes P450 in humans. European Journal of Clinical Pharmacology, 68(2), 213–217. 21870106 10.1007/s00228-011-1108-2 PMC4898966

[vms3660-bib-0005] Hamabe, W. , Maeda, T. , Fukazawa, Y. , Kumamoto, K. , Shang, L. Q. , Yamamoto, A. , Yamamoto, C. , Tokuyama, S. , & Kishioka, S. (2006). P‐glycoprotein ATPase activating effect of opioid analgesics and their P‐glycoprotein‐dependent antinociception in mice. Pharmacology, Biochemistry, and Behavior, 85(3), 629–636. 10.1016/j.pbb.2006.10.018 17134744

[vms3660-bib-0006] Handschin, C. , Podvinec, M. , & Meyer, U. A. (2000). CXR, a chicken xenobiotic‐sensing orphan nuclear receptor, is related to both mammalian pregnane X receptor (PXR) and constitutive androstane receptor (CAR). Proceedings of the National Academy of Sciences of the United States of America, 97(20), 10769–10774. 11005856 10.1073/pnas.97.20.10769 PMC27098

[vms3660-bib-0007] Handschin, C. , Podvinec, M. , Stöckli, J. , Hoffmann, K. , & Meyer, U. A. (2001). Conservation of signaling pathways of xenobiotic‐sensing orphan nuclear receptors, chicken xenobiotic receptor, constitutive androstane receptor, and pregnane X receptor, from birds to humans. Molecular Endocrinology, 15(9), 1571–1585. 11518807 10.1210/mend.15.9.0701

[vms3660-bib-0010] Lei, Z. , Liu, Q. , Yang, S. , Yang, B. , Khaliq, H. , Li, K. , Ahmed, S. , Sajid, A. , Zhang, B. , Chen, P. , Qiu, Y. , Cao, J. , & He, Q. (2018). PK‐pd integration modeling and cut off value of florfenicol against *Streptococcus suis* in pigs. Frontiers in Pharmacology, 9, 2. 29387013 10.3389/fphar.2018.00002 PMC5776115

[vms3660-bib-0011] Li, S. , Li, X. , Yuan, D. , Wang, B. , Yang, R. , Zhang, M. , Li, J. , & Zeng, F. (2017). Effects of paeoniflorin on the activities and mRNA expression of rat CYP1A2, CYP2C11 and CYP3A1 enzymes in vivo. Xenobiotica, 51, 961–967. 10.1080/00498254.2017.1404659 29160125

[vms3660-bib-0012] Li, S. , Li, X. , Yang, R. , Wang, B. , Li, J. , Cao, L. , Xiao, S. , & Huang, W. (2019). Effects of anemoside B4 on pharmacokinetics of florfenicol and mRNA expression of CXR, MDR1, CYP3A37 and UGT1E in broilers. The Journal of Veterinary Medical Science, 81(12), 1804–1809. 31611492 10.1292/jvms.19-0293 PMC6943327

[vms3660-bib-0013] Li, X. , Song, Y. , Wang, L. , Kang, G. , Wang, P. , Yin, H. , & Huang, H. (2021). A potential combination therapy of berberine hydrochloride with antibiotics against multidrug‐resistant *Acinetobacter baumannii* . Frontiers in Cellular and Infection Microbiology, 11, 660431. 33842399 10.3389/fcimb.2021.660431 PMC8027359

[vms3660-bib-0014] Livak, K. J. , & Schmittgen, T. D. (2001). Analysis of relative gene expression data using real‐time quantitative PCR and the 2(‐Delta Delta C(T)) Method. Methods, 25(4), 402–408. 11846609 10.1006/meth.2001.1262

[vms3660-bib-0015] Liu, C. , Wang, S. J. , Zhang, Q. , & Shao, Y. X. , 2015. Influence of three coccidiostats on the pharmacokinetics of florfenicol in rabbits. Experimental Animals, 64(1), 73–79. 25319758 10.1538/expanim.14-0064 PMC4329518

[vms3660-bib-0016] Liu, N. , Guo, M. , Mo, F. , Sun, Y. H. , Yuan, Z. , Cao, L. H. , & Jiang, S. X. (2012). Involvement of P‐glycoprotein and cytochrome P450 3A in the metabolism of florfenicol of rabbits. Journal of Veterinary Pharmacology and Therapeutics, 35(2), 202–205. 21615756 10.1111/j.1365-2885.2011.01310.x

[vms3660-bib-0017] Illambas, J. , Potter, T. , Sidhu, P. , Rycroft, A. N. , Cheng, Z. , & Lees, P. (2013). Pharmacodynamics of florfenicol for calf pneumonia pathogens. The Veterinary Record, 172(13), 340. 23482237 10.1136/vr.101155

[vms3660-bib-0018] Lo, A. , & Burckart, G. J. (1999). P‐glycoprotein and drug therapy in organ transplantation. Journal of Clinical Pharmacology, 39(10), 995–1005. 10516933 10.1177/00912709922011755

[vms3660-bib-0019] Luo, J. , Yan, D. , Yang, M. , Dong, X. , & Xiao, X. (2013). Multicomponent therapeutics of berberine alkaloids. Evidence‐Based Complementary and Alternative Medicine, 2013, 545898. 23634170 10.1155/2013/545898 PMC3619540

[vms3660-bib-0021] Pal, D. , & Mitra, A. K. (2006). MDR‐ and CYP3A4‐mediated drug‐drug interactions. Journal of Neuroimmune Pharmacology: The Official Journal of the Society on NeuroImmune Pharmacology, 1(3), 323–339. 18040809 10.1007/s11481-006-9034-2

[vms3660-bib-0022] Pérez, R. , Palma, C. , Drápela, C. , Sepulveda, M. , Espinoza, A. , & Peñailillo, A. K. (2015). Pharmacokinetics of florfenicol after intravenous administration in Escherichia coli lipopolysaccharide‐induced endotoxaemic sheep. Journal of Veterinary Pharmacology and Therapeutics, 38(2), 144–149. 25229993 10.1111/jvp.12160

[vms3660-bib-0023] Salmon, S. A. , & Watts, J. L. (2000). Minimum inhibitory concentration determinations for various antimicrobial agents against 1570 bacterial isolates from turkey poults. Avian Diseases, 44(1), 85–98. 10737648

[vms3660-bib-0024] Shen, Y. B. , Piao, X. S. , Kim, S. W. , Wang, L. , & Liu, P. (2010). The effects of berberine on the magnitude of the acute inflammatory response induced by *Escherichia coli* lipopolysaccharide in broiler chickens. Poultry Science, 89(1), 13–19. 10.3382/ps.2009-00243 20008797

[vms3660-bib-0025] Shi, C. , Li, M. , Muhammad, I. , Ma, X. , Chang, Y. , Li, R. , Li, C. , He, J. , & Liu, F. (2018). Combination of berberine and ciprofloxacin reduces multi‐resistant *Salmonella* strain biofilm formation by depressing mRNA expressions of *luxS*, *rpoE*, and *ompR* . Journal of Veterinary Science, 19(6), 808–816. 30304890 10.4142/jvs.2018.19.6.808 PMC6265579

[vms3660-bib-0026] Sidhu, P. , Rassouli, A. , Illambas, J. , Potter, T. , Pelligand, L. , Rycroft, A. , & Lees, P. (2014). Pharmacokinetic‐pharmacodynamic integration and modelling of florfenicol in calves. Journal of Veterinary Pharmacology and Therapeutics, 37(3), 231–242. 24341543 10.1111/jvp.12093

[vms3660-bib-0027] Slosky, L. M. , Thompson, B. J. , Sanchez‐Covarrubias, L. , Zhang, Y. , Laracuente, M. L. , Vanderah, T. W. , Ronaldson, P. T. , & Davis, T. P. (2013). Acetaminophen modulates P‐glycoprotein functional expression at the blood‐brain barrier by a constitutive androstane receptor‐dependent mechanism. Molecular Pharmacology, 84(5), 774–786. 24019224 10.1124/mol.113.086298 PMC3807077

[vms3660-bib-0038] Tang, X. , Xin, H. W. , Li, W. L. , & Ouyang, M. (2015). Study of the effects of Berberine on CYP3A4 and P‐gp in HepG2 cells and its mechanism in vitro. Chinese Journal of Clinical Pharmacology and Therapeutics, 20(1), 7–13 (In Chinese).

[vms3660-bib-0029] Tian, X. , Li, J. , Zamek‐Gliszczynski, M. J. , Bridges, A. S. , Zhang, P. , Patel, N. J. , Raub, T. J. , Pollack, G. M. , & Brouwer, K. L. (2007). Roles of P‐glycoprotein, Bcrp, and Mrp2 in biliary excretion of spiramycin in mice. Antimicrobial Agents and Chemotherapy, 51(9), 3230–3234. 17576841 10.1128/AAC.00082-07 PMC2043193

[vms3660-bib-0030] Wang, G. Y. (2012). Priliminary interaction study of florfenicol with polyether ionophore coccidiostats in broiler chichensnary interaction study of florfenicol with polyether ionophore coccidiostats in broiler chickens. Nanjing Agricultural University, Nanjing. 96pp. (In Chinese)

[vms3660-bib-0031] Wang, G. Y. , Tu, P. , Chen, X. , Guo, Y. G. , & Jiang, S. X. (2013). Effect of three polyether ionophores on pharmacokinetics of florfenicol in male broilers. Journal of Veterinary Pharmacology and Therapeutics, 36(5), 494–501. 23067134 10.1111/jvp.12020

[vms3660-bib-0032] Wang, G. Y. , Zheng, H. H. , Zhang, K. Y. , Yang, F. , Kong, T. , Zhou, B. , & Jiang, S. X. (2018). The roles of cytochrome P450 and P‐glycoprotein in the pharmacokinetics of florfenicol in chickens. Iranian Journal of Veterinary Research, 19(1), 9–14. 29805456 PMC5960766

[vms3660-bib-0033] Watanabe, K. P. , Kawai, Y. K. , Ikenaka, Y. , Kawata, M. , Ikushiro, S. , Sakaki, T. , & Ishizuka, M. (2013). Avian cytochrome P450 (CYP) 1–3 family genes: isoforms, evolutionary relationships, and mRNA expression in chicken liver. PloS One, 8(9), e75689. 24098714 10.1371/journal.pone.0075689 PMC3786927

[vms3660-bib-0034] Xin, H. W. , Wu, X. C. , Li, Q. , Yu, A. R. , Zhong, M. Y. , Zhu, M. , & Liu, Y. Y. (2004). Effects of berberine chloride and coadministration with cyclosporin on CPY3A1 in rat liver and small intestine. Chinese Journal of Clinical Pharmacology and Therapeutics, 9(5), 565–568. (In Chinese)

[vms3660-bib-0035] Xin, H. W. , Tang, X. , Ouyang, M. , Zhong, J. X. , & Li, W. L. (2016). Effects of berberine on pharmacokinetics of midazolam and rhodamine 123 in rats in vivo. SpringerPlus, 5, 380. 27066387 10.1186/s40064-016-2013-z PMC4811842

[vms3660-bib-0036] Zhang, Y. , Guo, L. , Huang, J. , Sun, Y. , He, F. , Zloh, M. , & Wang, L. (2019). Inhibitory effect of berberine on broiler P‐glycoprotein expression and function: in situ and in vitro studies. International Journal of Molecular Sciences, 20(8), 1966. 10.3390/ijms20081966 PMC6515058 31013627

